# Myristic acid hitchhiking on sigma-1 receptor to fend off neurodegeneration

**DOI:** 10.14800/rci.1114

**Published:** 2016

**Authors:** Jenna Ciesielski, Tsung-Ping Su, Shang-Yi Tsai

**Affiliations:** Cellular Pathobiology Section, Integrative Neuroscience Research Branch, Intramural Research Program, National Institute on Drug Abuse, National Institutes of Health, DHHS, Baltimore, Maryland 21224, USA

## Abstract

Neurodegenerative diseases are linked to tauopathy as a result of cyclin dependent kinase 5 (cdk5) binding to its p25 activator instead of its p35 activator and becoming over-activated. The overactive complex stimulates the hyperphosphorylation of tau proteins, leading to neurofibrillary tangles (NFTs) and stunting axon growth and development. It is known that the sigma-1 receptor (Sig-1R), an endoplasmic reticulum chaperone, can be involved in axon growth by promoting neurite sprouting through nerve growth factor (NGF) and tropomyosin receptor kinase B (TrkB)^[1, 2]^. It has also been previously demonstrated that a Sig-1R deficiency impairs the process of neurogenesis by causing a down-regulation of N-methyl-D-aspartate receptors (NMDARs)^[3]^. The recent study by Tsai *et al*. sought to understand the relationship between Sig-1R and tauopathy^[4]^. It was discovered that the Sig-1R helps maintain proper tau phosphorylation and axon development by facilitating p35 myristoylation and promoting p35 turnover. Neurons that had the Sig-1R knocked down exhibited shortened axons and higher levels of phosphorylated tau proteins compared to control neurons. Here we discuss these recent findings on the role of Sig-1R in tauopathy and highlight the newly presented physiological consequences of the Sig-1R-lipid interaction, helping to understand the close relationship between lipids and neurodegeneration.

Neurodegenerative and CNS diseases, such as Alzheimer’s disease and Parkinson’s disease, are in part caused by disturbances in proper axonal maintenance and can be recognized by a decrease in axonal length^[5–7]^. There are a variety of factors that can impact axon length: for example, proteins such as glial cell-line derived neurotrophic factor (GDNF) and nerve growth factor (NGF) can influence axon length, branching, and growth kinetics^[8]^, and the expression of ADP-ribosylation factor nucleotide-binding site opener (ARNO) and ADP-ribosylation factor 6 (ARF6) can result in enhanced axonal extension via downstream activation of phosphatidyl-inositol-4-phosphate 5-Kinase α [PI(4)P 5-Kinase α]^[9]^. It has also been demonstrated that sphingolipid synthesis is necessary for axon growth^[10]^.

In normally functioning neurons, tau proteins stabilize the structure of microtubules, contributing to proper axon growth^[11, 12]^. In contrast, in CNS disorders it is characteristic for tau proteins to be highly-phosphorylated and form neurofibrillary tangles (NFTs), often in aggregates known as paired helical filaments (PHFs)^[13]^. It has been proposed that hyperphosphorylation causes a functional loss of tau, preventing it from interacting with or stabilizing microtubules. This would result in axonal microtubules becoming destabilized and depolymerized and could cause neurons to degenerate^[14]^. It has also been suggested that abnormally phosphorylated tau proteins interact with normal tau proteins, making the latter unavailable to stabilize microtubules^[15]^. The kinases that phosphorylate tau proteins are generally divided into two categories: proline directed kinases and non-proline directed kinases^[16]^. Examples of proline directed kinases include GSK3B, cdk5, p38, MAP, and JNK, and examples of non-proline directed kinases include the tyrosine kinase fyn, MARK, PKA, PKC, and CK1^[16–19]^.

Important to this paper is the role of cyclin-dependent kinase 5 (cdk5), a proline directed kinase, in maintaining proper function of axonal maintenance by phosphorylating tau proteins. Cdk5 can be activated by p35 or p25^[20–25]^. These two activators cause different responses: p35 causes “beneficial” activation of cdk5, whereas p25 causes “abnormal” activation of cdk5. P35 has a relatively short half-life; there exists a negative feedback loop in which the activity of the p35/cdk5 kinase complex leads to autophosphorylation and degradation of p35 and therefore inactivation^[26]^. In adult neurons it is more common for p35 to be cleaved by calpain into p25^[27–29]^. P25 has a longer half-life than p35, so upon cleavage, p25 activates cdk5 and allows the complex to remain activated longer. In addition to prolonging activation of cdk5, p25 induces aberrant activation by releasing the complex from the membrane and allowing it to access additional substrates^[30]^. This overactive cdk5 complex can cause the hyperphosphorylation of tau proteins that leads to NFTs.

The study led by Tsai *et al*. examined the role of the Sig-1R, an endoplasmic reticulum (ER) chaperone, in the process of tauopathy^[4]^. Tsai and colleagues ultimately learned that the Sig-1R associates with myristic acid, promoting p35 turnover and regulating tau phosphorylation. To confirm the hypothesis that the Sig-1R is involved in regulating tau phosphorylation, Tsai *et al*. first transfected neurons with Sig-1R siRNA (siSig-1R) or control siRNA (SiCon) to verify that the Sig-1R is associated with axon development. When compared to the control group, it was seen that neurons transfected with siSig-1R resulted in reduced axon length. This supports the idea that the Sig-1R chaperone is involved in the regulation of axonal length and density. It was also discovered that diminished Sig-1R expression in neurons resulted in a noticeable accumulation of PHFs, which are indicative of hyperphosphorylated tau proteins and ultimately affect axon length.

When crude brain extracts from Sig-1R WT and KO mice were treated with CaCl_2_ to induce calpain activity, there was no difference in the cleavage of p35 to p25 between types of mice^[4]^. When taken together with data from treatments with the calpain inhibitor ALLM, these results show that the Sig-1R is not related to axonal length by affecting the conversion of p35 to p25 via calpain but rather by controlling the p35 degradation mainly through the proteasomal pathway.

Work by Patrick *et al*. demonstrated that p35 is more abundant than p25 in the membrane fraction, which may indicate that p35 is normally located at the membrane^[21]^. Asada *et al*. furthered this notion and revealed that myristoylation regulates the membrane association of p35^[31]^. Martin and Hayden recently reported that post-translational myristoylation (PTMyr) may not be limited to apoptosis and may play a role in cell survival, differentiation, and autophagy^[32]^. Tsai *et al*. determined that in the process of tauopathy, the Sig-1R binds myristic acid, which is used to myristoylate p35, and regulates the attachment of p35 to the membrane, perhaps by transferring myristic acid to p35^[4]^. Once p35 is myristoylated and bound to the membrane it can activate cdk5. Minegishi and colleagues found that both proteasomal degradation and calpain cleavage of p35 are stimulated by membrane association, which is in turn mediated via myristoylation of the N-terminal p10 region of p35. Therefore, when p35 is bound to the membrane the total turnover rate (by both degradation and cleavage) is greater than when p35 is not bound to the membrane^[30]^. The Sig-1R, by binding myristic acid, effectively helps balance the rate at which p35 is cleaved into p25 or degraded by proteasomes, serving thus as a modulator between the “normal” and “abnormal” activation of cdk5 and the regulation of axonal development.

By supplementing cells with exogenously added myristic acid, it was confirmed that myristic acid is important in regulating axon length and density^[4]^. In Sig-1R knockdown neurons, the addition of myristic acid eliminated irregular buildups of p35. Additionally, in WT and Sig-1R KO neurons, adding exogenous myristate not only amplified axon growth in the WT neurons but recovered the loss of axon length in KO neurons.

Several authors have previously reported on the relationship between Sig-1R and lipids. Results from Hayashi and Su indicate that the Sig-1R regulates the dynamics and compartmentalization of lipids on the ER^[33]^. Hayashi and Fujimoto stated that the Sig-1R is located at the MAM at specific ceramide- and cholesterol-rich lipid microdomains and that these lipid raft microdomains play a role in the distribution of Sig-1R^[34]^. When these sets of data are analyzed together they appear to indicate a seemingly reciprocal regulating relationship between the Sig-1R and lipids. On the one hand, it was found that changing the lipid membrane composition results in the translocation of Sig-1R, and it was thus proposed that the microdomains are used to anchor the Sig-1R to a location^[34]^. On the other hand, Palmer *et al*. provided evidence that in breast cancer cell lines the Sig-1R helps model and stabilize lipid rafts by binding to and inserting cholesterol into the membrane^[35]^. Slightly relevant to this relationship is a report that demonstrated that the Sig-1R associates with Insig in a 25-hydroxycholesterol-dependent manner to form an ER associated degradation (ERAD) system at the membrane and that the degradation of the sphingolipid enzyme CGalT is regulated by this ERAD system possibly through an interaction between CGalT and sterols^[36]^. Although those previous studies have shown that Sig-1Rs are interacting with the lipids, this paper by Tsai *et al*. reported for the first time on the physiological significance of the Sig-1R-lipid interaction^[4]^. Thus, the new finding of Tsai *et al*. suggests that the Sig-1R apparently provides the myristic acid, by means of myristic acid “hitchhiking” on the Sig-1R that allows p35 to bind to the lipid membrane where p35 can accomplish the balanced or homeostatic activation of cdk5. This ultimately results in the regulation of normal axonal growth and maintenance.
